# Supplementary material for: Multi-scale wastewater surveillance at a Bangkok tertiary care hospital: A potential sentinel site for real-time COVID-19 surveillance at hospital and national levels
Source: PLOS Glob Public Health. 2025 Apr 8;5(4):e0004256. doi: 10.1371/journal.pgph.0004256 (PMC11978038; doi:10.1371/journal.pgph.0004256)
Supplement: S1 Table — (DOCX) [file pgph.0004256.s001.docx]

**S1 Table. Hospital Twice Monthly COVID-19 Patient Case Report.**

| **Reporting Period** | | |  |  |
| --- | --- | --- | --- | --- |
| **Start Date** | | **End Date** | **Patient Cases** |  |
| 2022-07-01 | 2022-07-15 | | 1233 | |
| 2022-07-16 | 2022-07-31 | | 1071 | |
| 2022-08-01 | 2022-08-15 | | 1101 | |
| 2022-08-16 | 2022-08-31 | | 848 | |
| 2022-09-01 | 2022-09-15 | | 507 | |
| 2022-09-16 | 2022-09-30 | | 368 | |
| 2022-10-01 | 2022-10-15 | | 288 | |
| 2022-10-16 | 2022-10-31 | | 507 | |
| 2022-11-01 | 2022-11-15 | | 631 | |
| 2022-11-16 | 2022-11-30 | | 588 | |
| 2022-12-01 | 2022-12-15 | | 631 | |
| 2022-12-16 | 2022-12-31 | | 366 | |
| 2023-01-01 | 2023-01-15 | | 191 | |
| 2023-01-16 | 2023-01-31 | | 88 | |
| 2023-02-01 | 2023-02-15 | | 53 | |
| 2023-02-16 | 2023-02-28 | | 40 | |
| 2023-03-01 | 2023-03-15 | | 49 | |
| 2023-03-16 | 2023-03-31 | | 91 | |
| 2023-04-01 | 2023-04-15 | | 141 | |
| 2023-04-16 | 2023-04-30 | | 698 | |
| 2023-05-01 | 2023-05-15 | | 833 | |
| 2023-05-16 | 2023-05-31 | | 817 | |
