# Supplementary material for: Multi-scale wastewater surveillance at a Bangkok tertiary care hospital: A potential sentinel site for real-time COVID-19 surveillance at hospital and national levels
Source: PLOS Glob Public Health. 2025 Apr 8;5(4):e0004256. doi: 10.1371/journal.pgph.0004256 (PMC11978038; doi:10.1371/journal.pgph.0004256)
Supplement: S2 Table — (DOCX) [file pgph.0004256.s002.docx]

**S2 Table. Hospital Weekly COVID-19 Personnel Case Report.**

| **Reporting Period** | |  |
| --- | --- | --- |
| **Start Date** | **End Date** | **Personnel Cases** |
| 2022-06-27 | 2022-07-03 | 177 |
| 2022-07-04 | 2022-07-10 | 249 |
| 2022-07-11 | 2022-07-17 | 145 |
| 2022-07-18 | 2022-07-24 | 168 |
| 2022-07-25 | 2022-07-31 | 164 |
| 2022-08-01 | 2022-08-07 | 142 |
| 2022-08-08 | 2022-08-14 | 110 |
| 2022-08-15 | 2022-08-21 | 132 |
| 2022-08-22 | 2022-08-28 | 93 |
| 2022-08-29 | 2022-09-04 | 78 |
| 2022-09-05 | 2022-09-11 | 65 |
| 2022-09-12 | 2022-09-18 | 39 |
| 2022-09-19 | 2022-09-25 | 39 |
| 2022-09-26 | 2022-10-02 | 33 |
| 2022-10-03 | 2022-10-09 | 34 |
| 2022-10-10 | 2022-10-16 | 41 |
| 2022-10-17 | 2022-10-23 | 68 |
| 2022-10-24 | 2022-10-30 | 100 |
| 2022-10-31 | 2022-11-06 | 70 |
| 2022-11-07 | 2022-11-13 | 92 |
| 2022-11-14 | 2022-11-20 | 87 |
| 2022-11-21 | 2022-11-27 | 96 |
| 2022-11-28 | 2022-12-04 | 107 |
| 2022-12-05 | 2022-12-11 | 83 |
| 2022-12-12 | 2022-12-18 | 120 |
| 2022-12-19 | 2022-12-25 | 62 |
| 2022-12-26 | 2023-01-01 | 64 |
| 2023-01-02 | 2023-01-08 | 56 |
| 2023-01-09 | 2023-01-15 | 21 |
| 2023-01-16 | 2023-01-22 | 14 |
| 2023-01-23 | 2023-01-29 | 9 |
| 2023-01-30 | 2023-02-05 | 8 |
| 2023-02-06 | 2023-02-12 | 6 |
| 2023-02-13 | 2023-02-19 | 7 |
| 2023-02-20 | 2023-02-26 | 7 |
| 2023-02-27 | 2023-03-05 | 3 |
| 2023-03-06 | 2023-03-12 | 11 |
| 2023-03-13 | 2023-03-19 | 6 |
| 2023-03-20 | 2023-03-26 | 13 |
| 2023-03-27 | 2023-04-02 | 14 |
| 2023-04-03 | 2023-04-09 | 22 |
| 2023-04-10 | 2023-04-16 | 35 |
| 2023-04-17 | 2023-04-23 | 113 |
| 2023-04-24 | 2023-04-30 | 104 |
| 2023-05-01 | 2023-05-07 | 75 |
| 2023-05-08 | 2023-05-14 | 126 |
| 2023-05-15 | 2023-05-21 | 129 |
| 2023-05-22 | 2023-05-28 | 95 |
| 2023-05-29 | 2023-06-04 | 77 |
