# Supplementary material for: Multi-scale wastewater surveillance at a Bangkok tertiary care hospital: A potential sentinel site for real-time COVID-19 surveillance at hospital and national levels
Source: PLOS Glob Public Health. 2025 Apr 8;5(4):e0004256. doi: 10.1371/journal.pgph.0004256 (PMC11978038; doi:10.1371/journal.pgph.0004256)
Supplement: S3 Table — (DOCX) [file pgph.0004256.s003.docx]

**S3 Table. WHO Thailand Weekly COVID-19 Case Report.**

| **Date Reported** | **New Cases** |
| --- | --- |
| 2022-07-03 | 15950 |
| 2022-07-10 | 14938 |
| 2022-07-17 | 13986 |
| 2022-07-24 | 16824 |
| 2022-07-31 | 14323 |
| 2022-08-07 | 15433 |
| 2022-08-14 | 14816 |
| 2022-08-21 | 13755 |
| 2022-08-28 | 12232 |
| 2022-09-04 | 12130 |
| 2022-09-11 | 9004 |
| 2022-09-18 | 5841 |
| 2022-09-25 | 4965 |
| 2022-10-02 | 3780 |
| 2022-10-09 | 2915 |
| 2022-10-16 | 2234 |
| 2022-10-23 | 2616 |
| 2022-10-30 | 2551 |
| 2022-11-06 | 2759 |
| 2022-11-13 | 3166 |
| 2022-11-20 | 3957 |
| 2022-11-27 | 4914 |
| 2022-12-04 | 4284 |
| 2022-12-11 | 3961 |
| 2022-12-18 | 3419 |
| 2022-12-25 | 2900 |
| 2023-01-01 | 2111 |
| 2023-01-08 | 997 |
| 2023-01-15 | 969 |
| 2023-01-22 | 627 |
| 2023-01-29 | 472 |
| 2023-02-05 | 252 |
| 2023-02-12 | 392 |
| 2023-02-19 | 203 |
| 2023-02-26 | 204 |
| 2023-03-05 | 147 |
| 2023-03-12 | 122 |
| 2023-03-19 | 178 |
| 2023-03-26 | 150 |
| 2023-04-02 | 167 |
| 2023-04-09 | 168 |
| 2023-04-16 | 435 |
| 2023-04-23 | 1088 |
| 2023-04-30 | 1811 |
| 2023-05-07 | 1699 |
| 2023-05-14 | 2356 |
| 2023-05-21 | 2632 |
| 2023-05-28 | 2970 |
