# Supplementary material for: Multi-scale wastewater surveillance at a Bangkok tertiary care hospital: A potential sentinel site for real-time COVID-19 surveillance at hospital and national levels
Source: PLOS Glob Public Health. 2025 Apr 8;5(4):e0004256. doi: 10.1371/journal.pgph.0004256 (PMC11978038; doi:10.1371/journal.pgph.0004256)
Supplement: S4 Table — (DOCX) [file pgph.0004256.s004.docx]

**S4 Table. GISAID Accession Numbers of SARS-CoV-2 Genomic Surveillance Data in the Selected Hospital.**

|  |  | **Accession Number** | |
| --- | --- | --- | --- |
| **Date Collected** | **Clade** | **GISAID** | **GenBank** |
| 2022-07-01 | 22A (BA.4) | EPI_ISL_16259206 | |
| 2022-07-06 | 22B (BA.5) | EPI_ISL_16259207 | |
| 2022-07-12 | 22B (BA.5) | EPI_ISL_16259208 | |
| 2022-07-19 | 22B (BA.5) | EPI_ISL_16259209 | |
| 2022-07-20 | 22B (BA.5) | EPI_ISL_16259210 | |
| 2022-07-21 | 22B (BA.5) | EPI_ISL_16259211 | |
| 2022-07-25 | 21L (BA.2) | EPI_ISL_16259212 | |
| 2022-07-26 | 22B (BA.5) | EPI_ISL_16287694 | |
| 2022-08-01 | 22B (BA.5) | EPI_ISL_16259213 | |
| 2022-08-01 | 22B (BA.5) | EPI_ISL_16259214 | |
| 2022-08-03 | 21L (BA.2) | EPI_ISL_16259215 | |
| 2022-08-03 | 22B (BA.5) | EPI_ISL_16287695 | |
| 2022-08-04 | 22B (BA.5) | EPI_ISL_16287696 | |
| 2022-08-04 | 22B (BA.5) | EPI_ISL_16259216 | |
| 2022-08-05 | 22B (BA.5) | EPI_ISL_16259217 | |
| 2022-08-11 | 22B (BA.5) | EPI_ISL_16259218 | |
| 2022-08-15 | 22B (BA.5) | EPI_ISL_16259219 | |
| 2022-08-15 | 22B (BA.5) | EPI_ISL_16259220 | |
| 2022-08-16 | 22B (BA.5) | EPI_ISL_16259221 | |
| 2022-08-19 | 22B (BA.5) | EPI_ISL_16259222 | |
| 2022-08-29 | 22B (BA.5) | EPI_ISL_16259223 | |
| 2022-08-29 | 22B (BA.5) | EPI_ISL_16259224 | |
| 2022-08-31 | 22B (BA.5) | EPI_ISL_16259225 | |
| 2022-09-01 | 22B (BA.5) | EPI_ISL_16259226 | |
| 2022-09-05 | 22B (BA.5) | EPI_ISL_16287697 | |
| 2022-09-06 | 21L (BA.2) | EPI_ISL_16259227 | |
| 2022-09-06 | 21L (BA.2) | EPI_ISL_16259228 | |
| 2022-09-06 | 22B (BA.5) | EPI_ISL_16287698 | |
| 2022-09-07 | 22B (BA.5) | EPI_ISL_16259229 | |
| 2022-09-07 | 22B (BA.5) | EPI_ISL_16259230 | |
| 2022-09-12 | 22B (BA.5) | EPI_ISL_16259231 | |
| 2022-09-13 | 22B (BA.5) | EPI_ISL_16259232 | |
| 2022-09-13 | 22B (BA.5) | EPI_ISL_16259233 | |
| 2022-09-13 | 22B (BA.5) | EPI_ISL_16259234 | |
| 2022-09-14 | 22B (BA.5) | EPI_ISL_16259235 | |
| 2022-09-15 | 22B (BA.5) | EPI_ISL_16259236 | |
| 2022-09-19 | 22A (BA.4) | EPI_ISL_16259237 | |
| 2022-09-19 | 23C (CH.1.1) | EPI_ISL_16259238 | |
| 2022-09-21 | 22B (BA.5) | EPI_ISL_16259239 | |
| 2022-09-21 | 22D (BA.2.75) | EPI_ISL_16259240 | |
| 2022-09-22 | 22B (BA.5) | EPI_ISL_16287699 | |
| 2022-09-22 | 22B (BA.5) | EPI_ISL_16259241 | |
| 2022-09-26 | 22B (BA.5) | EPI_ISL_16259242 | |
| 2022-09-26 | 22B (BA.5) | EPI_ISL_16259243 | |
| 2022-09-26 | 22D (BA.2.75) | EPI_ISL_16259244 | |
| 2022-09-27 | 22B (BA.5) | EPI_ISL_16259245 | |
| 2022-09-28 | 22B (BA.5) | EPI_ISL_16259246 | |
| 2022-10-04 | 22B (BA.5) | EPI_ISL_16259247 | |
| 2022-10-05 | 22B (BA.5) | EPI_ISL_16287700 | |
| 2022-10-07 | 22B (BA.5) | EPI_ISL_16956952 | |
| 2022-10-17 | 22D (BA.2.75) | EPI_ISL_16259248 | |
| 2022-10-17 | 22B (BA.5) | EPI_ISL_16259249 | |
| 2022-10-17 | 22B (BA.5) | EPI_ISL_16259250 | |
| 2022-10-17 | 22B (BA.5) | EPI_ISL_16259251 | |
| 2022-10-19 | 22B (BA.5) | EPI_ISL_16259252 | |
| 2022-10-19 | 22D (BA.2.75) | EPI_ISL_16259253 | |
| 2022-10-20 | 22D (BA.2.75) | EPI_ISL_16956929 | |
| 2022-10-20 | 22D (BA.2.75) | EPI_ISL_16259254 | |
| 2022-10-21 | 22B (BA.5) | EPI_ISL_16259255 | |
| 2022-10-21 | 22D (BA.2.75) | EPI_ISL_16259256 | |
| 2022-10-21 | 22B (BA.5) | EPI_ISL_16259257 | |
| 2022-10-25 | 23C (CH.1.1) | EPI_ISL_16259258 | |
| 2022-10-25 | 22B (BA.5) | EPI_ISL_16259259 | |
| 2022-10-25 | 22B (BA.5) | EPI_ISL_16259260 | |
| 2022-10-25 | 22B (BA.5) | EPI_ISL_16259261 | |
| 2022-10-25 | 22D (BA.2.75) | EPI_ISL_16259262 | |
| 2022-10-25 | 22D (BA.2.75) | EPI_ISL_16259263 | |
| 2022-10-26 | 22D (BA.2.75) | EPI_ISL_16259264 | |
| 2022-10-27 | 22E (BQ.1) | EPI_ISL_16956930 | |
| 2022-10-27 | 22E (BQ.1) | EPI_ISL_16259265 | |
| 2022-10-27 | 22D (BA.2.75) | EPI_ISL_16956931 | |
| 2022-10-31 | 22D (BA.2.75) | EPI_ISL_16259266 | |
| 2022-10-31 | 22B (BA.5) | EPI_ISL_16956932 | |
| 2022-11-01 | 22F (XBB) | EPI_ISL_16259267 | |
| 2022-11-02 | 22B (BA.5) | EPI_ISL_16259268 | |
| 2022-11-02 | 22D (BA.2.75) | EPI_ISL_16259269 | |
| 2022-11-02 | 22B (BA.5) | EPI_ISL_16287701 | |
| 2022-11-04 | 22B (BA.5) | EPI_ISL_16956933 | |
| 2022-11-04 | 22D (BA.2.75) | EPI_ISL_16956934 | |
| 2022-11-07 | 22D (BA.2.75) | EPI_ISL_16956935 | |
| 2022-11-07 | 22D (BA.2.75) | EPI_ISL_16956936 | |
| 2022-11-09 | 22D (BA.2.75) | EPI_ISL_16956937 | |
| 2022-11-09 | 22B (BA.5) | EPI_ISL_17068927 | |
| 2022-11-09 | 22D (BA.2.75) | EPI_ISL_16956938 | |
| 2022-11-09 | 22E (BQ.1) | EPI_ISL_16956939 | |
| 2022-11-14 | 22D (BA.2.75) | EPI_ISL_16956946 | |
| 2022-11-14 | 22D (BA.2.75) | EPI_ISL_16956947 | |
| 2022-11-14 | 22D (BA.2.75) | EPI_ISL_16956948 | |
| 2022-11-14 | 22D (BA.2.75) | EPI_ISL_16956949 | |
| 2022-11-14 | 22D (BA.2.75) | EPI_ISL_16956950 | |
| 2022-11-15 | 22D (BA.2.75) | EPI_ISL_16956951 | |
| 2022-11-21 | 22B (BA.5) | EPI_ISL_16956940 | |
| 2022-11-21 | 22B (BA.5) | EPI_ISL_17068928 | |
| 2022-11-21 | 22D (BA.2.75) | EPI_ISL_16956941 | |
| 2022-11-21 | 23C (CH.1.1) | EPI_ISL_16956942 | |
| 2022-11-21 | 22D (BA.2.75) | EPI_ISL_16956943 | |
| 2022-11-22 | 22D (BA.2.75) | EPI_ISL_16956944 | |
| 2022-11-23 | 22D (BA.2.75) | EPI_ISL_16956945 | |
| 2022-11-23 | 22D (BA.2.75) | | PP301324 |
| 2022-11-23 | 22F (XBB) |  | PP301326 |
| 2022-11-24 | 22F (XBB) |  | PP301323 |
| 2022-11-25 | 22D (BA.2.75) | EPI_ISL_16956954 | |
| 2022-11-28 | 22F (XBB) |  | PP301325 |
| 2022-11-28 | 22D (BA.2.75) | EPI_ISL_16956955 | |
| 2022-11-28 | 22D (BA.2.75) | EPI_ISL_16956956 | |
| 2022-11-30 | 23C (CH.1.1) | EPI_ISL_16956957 | |
| 2022-12-14 | 22D (BA.2.75) | EPI_ISL_16956979 | |
| 2022-12-14 | 22D (BA.2.75) | EPI_ISL_16956980 | |
| 2022-12-14 | 22D (BA.2.75) | EPI_ISL_16956981 | |
| 2022-12-14 | 22D (BA.2.75) | EPI_ISL_16956982 | |
| 2022-12-14 | 22D (BA.2.75) | EPI_ISL_16956983 | |
| 2022-12-14 | 22D (BA.2.75) | EPI_ISL_16956984 | |
| 2022-12-16 | 22D (BA.2.75) | EPI_ISL_16956985 | |
| 2022-12-16 | 22D (BA.2.75) | EPI_ISL_16956986 | |
| 2022-12-16 | 22D (BA.2.75) | EPI_ISL_16956987 | |
| 2023-02-13 | Other Recombinant | | PP330153 |
| 2023-02-27 | 22D (BA.2.75) | | PP330154 |
| 2023-03-03 | 22B (BA.5) |  | PP330155 |
| 2023-03-20 | 23B (XBB.1.16) | | PP330156 |
| 2023-03-27 | 22D (BA.2.75) | | PP330157 |
| 2023-03-27 | 22F (XBB) |  | PP330158 |
| 2023-03-28 | 23A (XBB.1.5) | | PP330159 |
| 2023-04-10 | 23D (XBB.1.9) | | PP330160 |
| 2023-04-19 | 23B (XBB.1.16) | | PP330161 |
| 2023-04-18 | 23D (XBB.1.9) | | PP330162 |
| 2023-04-18 | 23D (XBB.1.9) | | PP330163 |
| 2023-04-18 | 23A (XBB.1.5) | | PP330164 |
| 2023-04-18 | 23A (XBB.1.5) | | PP330165 |
| 2023-04-19 | 23D (XBB.1.9) | | PP330166 |
| 2023-04-19 | 23A (XBB.1.5) | | PP330167 |
| 2023-04-20 | 23D (XBB.1.9) | | PP330168 |
| 2023-04-20 | 23A (XBB.1.5) | | PP330169 |
| 2023-04-24 | 23B (XBB.1.16) | | PP330170 |
| 2023-04-24 | 23D (XBB.1.9) | | PP330171 |
| 2023-04-24 | 23B (XBB.1.16) | | PP330172 |
| 2023-04-24 | 22B (BA.5) |  | PP330173 |
| 2023-05-01 | 23B (XBB.1.16) | | PP330174 |
| 2023-01-19 | 22D (BA.2.75) | EPI_ISL_17735483 | |
| 2023-05-02 | 23B (XBB.1.16) | | PP330175 |
| 2023-05-02 | 23B (XBB.1.16) | | PP330176 |
| 2023-05-03 | 23D (XBB.1.9) | | PP330177 |
| 2023-05-03 | 23B (XBB.1.16) | | PP330178 |
| 2023-05-11 | 23A (XBB.1.5) | | PP330179 |
| 2023-05-12 | 23B (XBB.1.16) | | PP330180 |
| 2023-05-18 | 23A (XBB.1.5) | | PP330181 |
| 2023-05-18 | 23B (XBB.1.16) | | PP330182 |
| 2023-05-23 | 23D (XBB.1.9) | | PP330183 |
| 2023-05-23 | 23B (XBB.1.16) | | PP330184 |
| 2023-05-24 | 22F (XBB) |  | PP330185 |
| 2023-05-25 | 23B (XBB.1.16) | | PP330186 |
| 2023-05-29 | 22F (XBB) |  | PP330187 |
| 2023-05-29 | 23D (XBB.1.9) | | PP330188 |
| 2023-05-30 | 23D (XBB.1.9) | | PP330189 |
| 2023-05-31 | 23B (XBB.1.16) | | PP330190 |
| 2023-05-21 | 23A (XBB.1.5) | | PP838530 |
| 2023-05-15 | 23B (XBB.1.16) | | PP838529 |
| 2023-05-19 | 23A (XBB.1.5) | | PP838528 |
| 2023-05-20 | 23B (XBB.1.16) | | PP838527 |
| 2023-05-29 | 23B (XBB.1.16) | | PP838526 |
| 2023-05-28 | 23E (XBB.2.3) | | PP838525 |
| 2023-05-30 | 23B (XBB.1.16) | | PP838524 |
| 2023-05-01 | 23B (XBB.1.16) | | PQ106951 |
| 2023-05-01 | 23E (XBB.2.3) | | PQ106952 |
| 2023-05-01 | 23B (XBB.1.16) | | PQ106953 |
| 2023-05-01 | 23A (XBB.1.5) | | PQ106954 |
| 2023-05-02 | 23D (XBB.1.9) | | PQ106955 |
| 2023-05-08 | 23D (XBB.1.9) | | PQ106956 |
| 2023-05-18 | 23D (XBB.1.9) | | PQ106957 |
| 2023-05-18 | 23B (XBB.1.16) | | PQ106958 |
| 2023-05-29 | 23B (XBB.1.16) | | PQ106959 |
| 2023-05-30 | 23B (XBB.1.16) | | PQ106960 |
| 2023-05-31 | 23A (XBB.1.5) | | PQ106961 |
| 2023-05-16 | 23D (XBB.1.9) | | PP446566 |
| 2023-05-16 | 23B (XBB.1.16) | | PP446565 |
| 2023-05-11 | 23A (XBB.1.5) | | PP446564 |
| 2023-05-15 | 23D (XBB.1.9) | | PP446563 |
| 2023-05-11 | 23F (EG.5.1) |  | PP446562 |
| 2023-05-12 | 23D (XBB.1.9) | | PP446561 |
| 2023-05-14 | 23B (XBB.1.16) | | PP446560 |
| 2023-05-14 | 23D (XBB.1.9) | | PP446559 |
| 2023-05-14 | 23F (EG.5.1) |  | PP446558 |
| 2023-05-15 | 23D (XBB.1.9) | | PP446557 |
| 2023-05-15 | 23B (XBB.1.16) | | PP446556 |
| 2023-05-18 | 23D (XBB.1.9) | | PP446555 |
| 2023-05-18 | 23B (XBB.1.16) | | PP446554 |
| 2023-05-20 | 23A (XBB.1.5) | | PP446553 |
| 2023-05-19 | 23D (XBB.1.9) | | PP446552 |
| 2023-05-19 | 23D (XBB.1.9) | | PP446551 |
| 2023-05-21 | 23A (XBB.1.5) | | PP446570 |
| 2023-05-21 | 23D (XBB.1.9) | | PP446571 |
| 2023-05-21 | 23A (XBB.1.5) | | PP446572 |
| 2023-05-21 | 23B (XBB.1.16) | | PP446573 |
| 2023-05-19 | 23B (XBB.1.16) | | PP446574 |
| 2023-05-22 | 23B (XBB.1.16) | | PP446575 |
| 2023-05-22 | 23B (XBB.1.16) | | PP446576 |
| 2023-05-24 | 23A (XBB.1.5) | | PP446577 |
| 2023-05-24 | 23D (XBB.1.9) | | PP446578 |
| 2023-05-24 | 23A (XBB.1.5) | | PP446579 |
| 2023-05-24 | 23A (XBB.1.5) | | PP446580 |
| 2023-05-24 | 23D (XBB.1.9) | | PP446581 |
| 2023-05-26 | 23D (XBB.1.9) | | PP446582 |
| 2023-05-28 | 23A (XBB.1.5) | | PP446583 |
| 2023-05-28 | 23B (XBB.1.16) | | PP446584 |
| 2023-05-29 | 23B (XBB.1.16) | | PP446585 |
| 2023-05-29 | 23D (XBB.1.9) | | PP446586 |
