# Supplementary material for: Multi-scale wastewater surveillance at a Bangkok tertiary care hospital: A potential sentinel site for real-time COVID-19 surveillance at hospital and national levels
Source: PLOS Glob Public Health. 2025 Apr 8;5(4):e0004256. doi: 10.1371/journal.pgph.0004256 (PMC11978038; doi:10.1371/journal.pgph.0004256)
Supplement: S5 Table — (DOCX) [file pgph.0004256.s005.docx]

**S5 Table. Raw RT-PCR Wastewater Data.**

|  | **Ct Value** | | |  |  |
| --- | --- | --- | --- | --- | --- |
| **Date Collected** | **E** | **ORF1ab** | **N** | **SARS-CoV-2 Detection** | **Sample Site** |
| 2022-07-07 | 29.03 | 28.34 | 26.36 | Detected | B1 |
| 2022-07-07 | 29.37 | 30.31 | 27.81 | Detected | B2 |
| 2022-07-07 | 23.18 | 21.27 | 23.08 | Detected | B3 |
| 2022-07-07 | 30.55 | 32.19 | 30.82 | Detected | B4 |
| 2022-07-07 | 27.47 | 25.82 | 27.47 | Detected | C1 |
| 2022-07-07 | 30.79 | 29.22 | 30.70 | Detected | TCe |
| 2022-07-12 |  | 32.47 | 30.22 | Detected | B1 |
| 2022-07-12 | 27.11 | 27.32 | 25.79 | Detected | B2 |
| 2022-07-12 | 23.92 | 24.05 | 22.07 | Detected | B3 |
| 2022-07-12 | 27.03 | 27.44 | 25.95 | Detected | B4 |
| 2022-07-12 | 26.06 | 25.44 | 23.75 | Detected | C1 |
| 2022-07-12 | 31.14 | 30.22 | 29.15 | Detected | TCe |
| 2022-07-19 | 26.72 | 26.65 | 24.85 | Detected | B1 |
| 2022-07-19 | 27.14 | 27.27 | 25.08 | Detected | B2 |
| 2022-07-19 | 22.69 | 22.61 | 20.66 | Detected | B3 |
| 2022-07-19 | 27.90 | 27.60 | 26.33 | Detected | B4 |
| 2022-07-19 | 27.51 | 26.53 | 26.53 | Detected | C1 |
| 2022-07-19 | 29.87 | 27.35 | 30.24 | Detected | TCe |
| 2022-07-26 | 29.31 | 28.90 | 27.12 | Detected | B1 |
| 2022-07-26 | 29.29 | 29.04 | 27.05 | Detected | B2 |
| 2022-07-26 | 26.81 | 26.35 | 24.28 | Detected | B3 |
| 2022-07-26 | 31.37 | 31.13 | 28.94 | Detected | B4 |
| 2022-07-26 | 30.24 | 29.47 | 28.04 | Detected | C1 |
| 2022-07-26 | 25.32 | 24.67 | 22.59 | Detected | TCe |
| 2022-08-02 | 27.27 | 26.85 | 27.27 | Detected | B1 |
| 2022-08-02 | 29.5 | 31.03 | 28.21 | Detected | B2 |
| 2022-08-02 | 26.08 | 25.98 | 24.38 | Detected | B3 |
| 2022-08-02 | 27.57 | 27.35 | 25.69 | Detected | B4 |
| 2022-08-02 | 33.00 | 33.37 |  | Detected | C1 |
| 2022-08-02 | 29.19 | 28.51 | 27.29 | Detected | TCe |
| 2022-08-09 | 30.74 | 30.15 | 28.37 | Detected | B1 |
| 2022-08-09 | 29.73 | 28.78 | 27.16 | Detected | B2 |
| 2022-08-09 | 23.25 | 22.13 | 21.04 | Detected | B3 |
| 2022-08-09 | 31.04 | 30.41 | 29.60 | Detected | B4 |
| 2022-08-09 | 28.39 | 27.45 | 26.29 | Detected | C1 |
| 2022-08-09 | 33.87 | 32.54 | 30.16 | Detected | TCe |
| 2022-08-16 | 28.39 | 28.10 | 26.02 | Detected | B1 |
| 2022-08-16 | 27.64 | 27.22 | 24.58 | Detected | B2 |
| 2022-08-16 | 27.89 | 27.11 | 25.35 | Detected | B3 |
| 2022-08-16 | 30.83 | 29.27 | 28.56 | Detected | B4 |
| 2022-08-16 | 27.45 | 26.56 | 24.99 | Detected | C1 |
| 2022-08-16 |  |  |  | Not detected | TCe |
| 2022-08-23 | 32.16 | 31.36 | 29.29 | Detected | B1 |
| 2022-08-23 | 29.77 | 28.64 | 27.60 | Detected | B2 |
| 2022-08-23 | 27.45 | 26.65 | 25.17 | Detected | B3 |
| 2022-08-23 | 29.36 | 29.16 | 27.04 | Detected | B4 |
| 2022-08-23 | 30.45 | 29.56 | 28.07 | Detected | C1 |
| 2022-08-23 | 31.73 | 32.84 | 30.24 | Detected | TCe |
| 2022-08-30 | 31.80 | 31.84 | 28.74 | Detected | B1 |
| 2022-08-30 | 31.99 | 32.91 | 31.38 | Detected | B2 |
| 2022-08-30 | 34.01 | 32.37 | 30.04 | Detected | B3 |
| 2022-08-30 | 32.66 | 32.09 | 29.50 | Detected | B4 |
| 2022-08-30 |  |  |  | Not detected | C1 |
| 2022-08-30 |  | 33.48 | 31.59 | Detected | TCe |
| 2022-09-06 | 30.38 | 31.06 | 28.72 | Detected | B1 |
| 2022-09-06 | 30.32 | 29.5 | 28.33 | Detected | B2 |
| 2022-09-06 | 24.76 | 24.04 | 22.68 | Detected | B3 |
| 2022-09-06 | 32.14 | 33.01 | 30.88 | Detected | B4 |
| 2022-09-06 | 30.50 | 29.48 | 28.73 | Detected | C1 |
| 2022-09-06 |  | 33.11 | 32.27 | Detected | TCe |
| 2022-09-13 | 30.04 | 29.57 | 28.2 | Detected | B1 |
| 2022-09-13 | 29.03 | 29.68 | 27.48 | Detected | B2 |
| 2022-09-13 | 25.68 | 25.07 | 24.03 | Detected | B3 |
| 2022-09-13 | 31.19 | 30.92 | 29.07 | Detected | B4 |
| 2022-09-13 | 30.47 | 30.27 | 28.05 | Detected | C1 |
| 2022-09-13 | 32.27 | 36.22 | 30.01 | Detected | TCe |
| 2022-09-20 | 30.10 | 28.81 | 27.11 | Detected | B1 |
| 2022-09-20 | 27.58 | 26.53 | 25.27 | Detected | B2 |
| 2022-09-20 | 28.21 | 27.5 | 26.16 | Detected | B3 |
| 2022-09-20 | 30.62 | 30.09 | 28.31 | Detected | B4 |
| 2022-09-20 | 29.65 | 28.48 | 27.24 | Detected | C1 |
| 2022-09-20 |  | 33.11 | 32.66 | Detected | TCe |
| 2022-09-27 | 30.32 | 30.82 | 29.01 | Detected | B1 |
| 2022-09-27 | 32.26 | 32.36 | 29.71 | Detected | B2 |
| 2022-09-27 | 26.06 | 26.84 | 24.39 | Detected | B3 |
| 2022-09-27 | 28.36 | 27.36 | 26.38 | Detected | B4 |
| 2022-09-27 | 29.66 | 30.39 | 28.86 | Detected | C1 |
| 2022-09-27 | 34.21 |  | 32.72 | Detected | TCe |
| 2022-10-04 |  |  |  | Not detected | B1 |
| 2022-10-04 | 31.11 | 32.01 | 28.89 | Detected | B2 |
| 2022-10-04 | 32.07 | 32.77 | 28.49 | Detected | B3 |
| 2022-10-04 | 27.51 | 26.77 | 25.59 | Detected | B4 |
| 2022-10-04 | 30.45 | 31.39 | 27.82 | Detected | C1 |
| 2022-10-04 | 34.75 |  | 32.98 | Detected | TCe |
| 2022-10-11 | 30.84 | 27.20 | 27.05 | Detected | B1 |
| 2022-10-11 | 30.48 | 28.06 | 27.07 | Detected | B2 |
| 2022-10-11 | 26.39 | 25.49 | 24.04 | Detected | B3 |
| 2022-10-11 | 30.41 | 30.29 | 27.77 | Detected | B4 |
| 2022-10-11 | 32.01 | 29.49 | 29.49 | Detected | C1 |
| 2022-10-11 |  |  |  | Not detected | TCe |
| 2022-10-18 | 28.32 | 28.45 | 26.09 | Detected | B1 |
| 2022-10-18 | 24.03 | 23.50 | 21.50 | Detected | B2 |
| 2022-10-18 | 24.49 | 24.12 | 22.22 | Detected | B3 |
| 2022-10-18 | 29.73 | 26.97 | 26.51 | Detected | B4 |
| 2022-10-18 | 29.62 | 27.12 | 27.14 | Detected | C1 |
| 2022-10-18 |  | 30.38 | 30.13 | Detected | TCe |
| 2022-10-25 | 28.76 | 27.91 | 26.47 | Detected | B1 |
| 2022-10-25 | 30.34 | 28.72 | 27.66 | Detected | B2 |
| 2022-10-25 | 21.96 | 21.54 | 19.42 | Detected | B3 |
| 2022-10-25 | 30.22 | 29.14 | 27.45 | Detected | B4 |
| 2022-10-25 | 28.01 | 26.72 | 25.30 | Detected | C1 |
| 2022-10-25 | 31.04 | 28.84 | 29.55 | Detected | TCe |
| 2022-11-01 | 26.95 | 26.50 | 24.33 | Detected | B1 |
| 2022-11-01 | 28.45 | 28.41 | 26.33 | Detected | B2 |
| 2022-11-01 | 26.24 | 25.97 | 23.65 | Detected | B3 |
| 2022-11-01 | 26.52 | 26.33 | 24.11 | Detected | B4 |
| 2022-11-01 | 29.06 | 28.25 | 26.38 | Detected | C1 |
| 2022-11-01 |  | 32.00 | 31.27 | Detected | TCe |
| 2022-11-08 | 30.75 | 29.30 | 28.13 | Detected | B1 |
| 2022-11-08 | 26.66 | 26.06 | 24.70 | Detected | B2 |
| 2022-11-08 | 20.23 | 19.45 | 17.36 | Detected | B3 |
| 2022-11-08 | 32.10 | 29.87 | 29.59 | Detected | B4 |
| 2022-11-08 | 26.98 | 26.07 | 24.04 | Detected | C1 |
| 2022-11-08 | 24.97 | 24.00 | 22.51 | Detected | TCe |
| 2022-11-15 | 28.09 | 27.55 | 26.00 | Detected | B1 |
| 2022-11-15 | 28.75 | 28.23 | 26.40 | Detected | B2 |
| 2022-11-15 | 24.07 | 23.5 | 21.26 | Detected | B3 |
| 2022-11-15 | 31.13 | 29.88 | 27.89 | Detected | B4 |
| 2022-11-15 | 29.75 | 30.02 | 27.36 | Detected | C1 |
| 2022-11-15 | 32.70 | 30.74 | 29.2 | Detected | TCe |
| 2022-11-22 | 27.87 | 28.18 | 26.12 | Detected | B1 |
| 2022-11-22 | 26.41 | 26.58 | 24.03 | Detected | B2 |
| 2022-11-22 | 29.13 | 28.71 | 27.00 | Detected | B3 |
| 2022-11-22 | 27.05 | 28.01 | 24.57 | Detected | B4 |
| 2022-11-22 | 30.88 | 30.16 | 30.02 | Detected | C1 |
| 2022-11-22 | 31.75 | 31.54 | 29.96 | Detected | TCe |
| 2022-11-29 | 31.22 | 31.03 | 29.63 | Detected | B1 |
| 2022-11-29 | 30.54 | 30.32 | 28.34 | Detected | B2 |
| 2022-11-29 | 30.49 | 31.18 | 29.00 | Detected | B3 |
| 2022-11-29 | 29.63 | 29.59 | 27.90 | Detected | B4 |
| 2022-11-29 | 31.04 | 32.27 | 30.32 | Detected | C1 |
| 2022-11-29 |  |  |  | Not detected | TCe |
| 2022-12-06 | 32.31 | 32.38 | 30.07 | Detected | B1 |
| 2022-12-06 | 27.94 | 27.96 | 26.98 | Detected | B2 |
| 2022-12-06 | 27.21 | 27.95 | 24.86 | Detected | B3 |
| 2022-12-06 | 30.10 | 29.25 | 28.45 | Detected | B4 |
| 2022-12-06 | 30.78 | 30.54 | 29.09 | Detected | C1 |
| 2022-12-06 |  |  |  | Not detected | TCe |
| 2022-12-06 | 26.93 | 28.21 | 26.26 | Detected | B5 |
| 2022-12-06 | 30.50 | 30.07 | 28.93 | Detected | B6 |
| 2022-12-06 | 27.46 | 25.33 | 24.64 | Detected | C2 |
| 2022-12-06 | 27.59 | 23.09 | 23.20 | Detected | TCi |
| 2022-12-13 | 32.08 | 31.03 | 29.10 | Detected | B1 |
| 2022-12-13 | 29.89 | 27.99 | 27.01 | Detected | B2 |
| 2022-12-13 | 25.39 | 24.19 | 21.76 | Detected | B3 |
| 2022-12-13 | 28.75 | 27.95 | 25.27 | Detected | B4 |
| 2022-12-13 | 33.44 | 32.98 | 29.57 | Detected | C1 |
| 2022-12-13 |  |  |  | Not detected | TCe |
| 2022-12-13 | 30.14 | 28.61 | 27.22 | Detected | B5 |
| 2022-12-13 | 25.67 | 25.17 | 22.64 | Detected | B6 |
| 2022-12-13 | 29.20 | 28.57 | 26.30 | Detected | C2 |
| 2022-12-13 | 28.27 | 26.74 | 24.68 | Detected | TCi |
| 2022-12-20 | 30.26 | 29.47 | 26.54 | Detected | B1 |
| 2022-12-20 | 31.30 | 30.27 | 28.29 | Detected | B2 |
| 2022-12-20 | 23.30 | 22.42 | 20.33 | Detected | B3 |
| 2022-12-20 | 29.97 | 28.25 | 25.85 | Detected | B4 |
| 2022-12-20 | 33.22 | 31.31 | 29.62 | Detected | C1 |
| 2022-12-20 |  |  |  | Not detected | TCe |
| 2022-12-20 | 30.37 | 30.41 | 27.47 | Detected | B5 |
| 2022-12-20 | 29.03 | 28.25 | 26.53 | Detected | B6 |
| 2022-12-20 | 22.01 | 21.06 | 19.15 | Detected | C2 |
| 2022-12-20 | 28.63 | 27.20 | 25.27 | Detected | TCi |
| 2022-12-27 | 33.23 | 29.92 | 31.17 | Detected | B1 |
| 2022-12-27 | 32.55 | 31.85 | 28.84 | Detected | B2 |
| 2022-12-27 | 27.45 | 26.33 | 24.87 | Detected | B3 |
| 2022-12-27 | 28.59 | 27.67 | 25.97 | Detected | B4 |
| 2022-12-27 | 34.19 | 31.72 | 29.96 | Detected | C1 |
| 2022-12-27 |  |  |  | Not detected | TCe |
| 2022-12-27 | 25.11 | 23.99 | 21.59 | Detected | B5 |
| 2022-12-27 | 27.25 | 26.10 | 24.65 | Detected | B6 |
| 2022-12-27 | 27.54 | 26.00 | 24.21 | Detected | C2 |
| 2022-12-27 | 28.48 | 27.16 | 25.01 | Detected | TCi |
| 2023-01-03 | 28.16 | 27.50 | 25.58 | Detected | B1 |
| 2023-01-03 | 29.54 | 30.72 | 28.67 | Detected | B2 |
| 2023-01-03 | 29.02 | 27.73 | 26.31 | Detected | B3 |
| 2023-01-03 | 30.88 | 31.88 | 28.88 | Detected | B4 |
| 2023-01-03 | 31.64 | 30.88 | 29.04 | Detected | C1 |
| 2023-01-03 |  |  |  | Not detected | TCe |
| 2023-01-03 | 28.70 | 28.12 | 26.34 | Detected | B5 |
| 2023-01-03 | 27.12 | 26.79 | 24.45 | Detected | B6 |
| 2023-01-03 | 30.40 | 29.55 | 27.39 | Detected | C2 |
| 2023-01-03 | 27.98 | 26.80 | 24.96 | Detected | TCi |
| 2023-01-10 | 31.76 | 31.21 | 31.51 | Detected | B1 |
| 2023-01-10 | 30.91 | 32.07 | 29.23 | Detected | B2 |
| 2023-01-10 | 27.58 | 27.46 | 25.89 | Detected | B3 |
| 2023-01-10 | 30.56 |  | 30.02 | Detected | B4 |
| 2023-01-10 | 32.26 | 31.62 | 29.29 | Detected | C1 |
| 2023-01-10 | 34.03 |  | 32.65 | Detected | TCe |
| 2023-01-10 | 30.72 | 30.24 | 27.81 | Detected | B5 |
| 2023-01-10 | 28.49 | 28.27 | 25.91 | Detected | B6 |
| 2023-01-10 | 30.14 | 28.69 | 27.63 | Detected | C2 |
| 2023-01-10 | 30.10 | 29.61 | 26.96 | Detected | TCi |
| 2023-01-17 | 30.64 | 31.19 | 28.55 | Detected | B1 |
| 2023-01-17 | 27.34 | 27.05 | 25.39 | Detected | B2 |
| 2023-01-17 | 27.42 | 27.42 | 24.23 | Detected | B3 |
| 2023-01-17 |  |  |  | Not detected | B4 |
| 2023-01-17 |  |  |  | Not detected | C1 |
| 2023-01-17 | 32.40 |  | 30.96 | Detected | TCe |
| 2023-01-17 | 26.53 | 26.32 | 24.18 | Detected | B5 |
| 2023-01-17 |  | 31.87 | 32.43 | Detected | B6 |
| 2023-01-17 | 28.08 | 27.31 | 25.49 | Detected | C2 |
| 2023-01-17 | 33.06 | 31.10 | 30.34 | Detected | TCi |
| 2023-01-24 | 33.66 | 31.69 | 30.90 | Detected | B1 |
| 2023-01-24 | 31.29 | 30.13 | 29.07 | Detected | B2 |
| 2023-01-24 | 30.97 | 29.76 | 27.18 | Detected | B3 |
| 2023-01-24 | 31.96 | 30.33 | 29.00 | Detected | B4 |
| 2023-01-24 |  |  |  | Not detected | C1 |
| 2023-01-24 |  |  |  | Not detected | TCe |
| 2023-01-24 | 30.16 | 29.31 | 28.48 | Detected | B5 |
| 2023-01-24 | 31.28 | 30.91 | 28.73 | Detected | B6 |
| 2023-01-24 | 30.96 | 31.03 | 28.65 | Detected | C2 |
| 2023-01-24 | 30.72 | 29.44 | 28.23 | Detected | TCi |
| 2023-01-31 | 31.03 | 29.25 | 28.79 | Detected | B1 |
| 2023-01-31 |  |  |  | Not detected | B2 |
| 2023-01-31 | 28.62 | 28.41 | 26.10 | Detected | B3 |
| 2023-01-31 | 33.25 | 32.94 | 31.85 | Detected | B4 |
| 2023-01-31 | 33.39 | 33.12 |  | Detected | C1 |
| 2023-01-31 |  |  |  | Not detected | TCe |
| 2023-01-31 | 31.42 | 30.82 | 28.44 | Detected | B5 |
| 2023-01-31 |  | 32.07 | 32.74 | Detected | B6 |
| 2023-01-31 | 29.49 | 29.46 | 26.74 | Detected | C2 |
| 2023-01-31 | 28.95 | 28.09 | 26.60 | Detected | TCi |
| 2023-02-07 |  | 32.87 | 31.78 | Detected | B1 |
| 2023-02-07 |  |  |  | Not detected | B2 |
| 2023-02-07 | 31.97 | 31.94 | 30.02 | Detected | B3 |
| 2023-02-07 | 31.35 | 31.04 | 30.42 | Detected | B4 |
| 2023-02-07 | 33.14 |  | 32.72 | Detected | C1 |
| 2023-02-07 |  |  |  | Not detected | TCe |
| 2023-02-07 |  | 34.2 | 32.24 | Detected | B5 |
| 2023-02-07 |  |  |  | Not detected | B6 |
| 2023-02-07 | 33.75 |  | 31.95 | Detected | C2 |
| 2023-02-07 | 30.07 | 30.61 | 28.04 | Detected | TCi |
| 2023-02-14 |  |  |  | Not detected | B1 |
| 2023-02-14 |  |  |  | Not detected | B2 |
| 2023-02-14 | 33.10 | 32.65 | 33.24 | Detected | B3 |
| 2023-02-14 | 33.24 | 32.40 | 32.65 | Detected | B4 |
| 2023-02-14 |  |  |  | Not detected | C1 |
| 2023-02-14 |  |  |  | Not detected | TCe |
| 2023-02-14 |  | 33.47 | 33.48 | Detected | B5 |
| 2023-02-14 |  |  |  | Not detected | B6 |
| 2023-02-14 | 33.47 |  | 35.17 | Detected | C2 |
| 2023-02-14 | 33.10 | 32.40 | 32.07 | Detected | TCi |
| 2023-02-21 | 33.10 | 32.65 | 32.97 | Detected | B1 |
| 2023-02-21 |  | 32.96 | 32.47 | Detected | B2 |
| 2023-02-21 | 29.82 | 29.83 | 27.09 | Detected | B3 |
| 2023-02-21 | 33.35 | 32.07 | 29.79 | Detected | B4 |
| 2023-02-21 |  | 31.57 | 34.29 | Detected | C1 |
| 2023-02-21 |  |  |  | Not detected | TCe |
| 2023-02-21 |  | 34.45 | 33.92 | Detected | B5 |
| 2023-02-21 |  |  |  | Not detected | B6 |
| 2023-02-21 |  |  |  | Not detected | C2 |
| 2023-02-21 | 33.10 | 31.68 | 31.08 | Detected | TCi |
| 2023-02-28 | 28.14 | 27.65 | 26.20 | Detected | B1 |
| 2023-02-28 |  | 32.18 | 32.01 | Detected | B2 |
| 2023-02-28 | 34.09 | 32.11 |  | Detected | B3 |
| 2023-02-28 | 24.37 | 24.04 | 22.45 | Detected | B4 |
| 2023-02-28 |  |  |  | Not detected | C1 |
| 2023-02-28 | 34.22 | 33.42 |  | Detected | TCe |
| 2023-02-28 |  | 32.25 | 33.29 | Detected | B5 |
| 2023-02-28 | 24.99 | 25.53 | 23.16 | Detected | B6 |
| 2023-02-28 |  |  |  | Not detected | C2 |
| 2023-02-28 | 30.48 | 32.10 | 29.91 | Detected | TCi |
| 2023-03-07 | 33.19 |  | 32.49 | Detected | B1 |
| 2023-03-07 | 34.00 | 33.97 | 31.48 | Detected | B2 |
| 2023-03-07 |  |  |  | Not detected | B3 |
| 2023-03-07 |  |  |  | Not detected | B4 |
| 2023-03-07 | 33.99 |  | 31.57 | Detected | C1 |
| 2023-03-07 | 34.20 |  | 35.05 | Detected | TCe |
| 2023-03-07 | 33.92 | 31.18 | 31.08 | Detected | B5 |
| 2023-03-07 | 32.95 | 28.46 | 29.95 | Detected | B6 |
| 2023-03-07 |  | 31.89 | 31.62 | Detected | C2 |
| 2023-03-07 | 29.57 | 29.58 | 27.07 | Detected | TCi |
| 2023-03-14 |  |  |  | Not detected | B1 |
| 2023-03-14 | 32.24 | 31.25 | 29.4 | Detected | B2 |
| 2023-03-14 | 30.01 | 32.79 | 32.77 | Detected | B3 |
| 2023-03-14 |  |  |  | Not detected | B4 |
| 2023-03-14 |  |  |  | Not detected | C1 |
| 2023-03-14 |  |  |  | Not detected | TCe |
| 2023-03-14 | 33.03 | 31.26 | 30.14 | Detected | B5 |
| 2023-03-14 | 32.25 | 31.23 | 29.80 | Detected | B6 |
| 2023-03-14 |  |  |  | Not detected | C2 |
| 2023-03-14 | 26.86 | 27.68 | 28.44 | Detected | TCi |
| 2023-03-21 |  |  |  | Not detected | B1 |
| 2023-03-21 | 33.80 |  | 32.64 | Detected | B2 |
| 2023-03-21 |  |  |  | Not detected | B3 |
| 2023-03-21 | 29.41 | 30.48 | 29.00 | Detected | B4 |
| 2023-03-21 |  |  |  | Not detected | C1 |
| 2023-03-21 |  |  |  | Not detected | TCe |
| 2023-03-21 |  |  |  | Not detected | B5 |
| 2023-03-21 | 34.15 | 35.82 | 32.93 | Detected | B6 |
| 2023-03-21 |  |  |  | Not detected | C2 |
| 2023-03-21 | 30.26 | 29.84 | 27.69 | Detected | TCi |
| 2023-03-28 | 32.43 |  | 32.27 | Detected | B1 |
| 2023-03-28 |  | 32.93 | 32.16 | Detected | B2 |
| 2023-03-28 |  |  |  | Not detected | B3 |
| 2023-03-28 | 26.22 | 26.04 | 24.52 | Detected | B4 |
| 2023-03-28 |  |  |  | Not detected | C1 |
| 2023-03-28 |  |  |  | Not detected | TCe |
| 2023-03-28 | 32.88 | 32.60 | 30.28 | Detected | B5 |
| 2023-03-28 | 23.68 | 23.08 | 21.37 | Detected | B6 |
| 2023-03-28 | 34.11 |  | 32.45 | Detected | C2 |
| 2023-03-28 | 31.62 | 31.31 | 29.17 | Detected | TCi |
| 2023-04-04 |  |  |  | Not detected | B1 |
| 2023-04-04 |  |  |  | Not detected | B2 |
| 2023-04-04 |  |  |  | Not detected | B3 |
| 2023-04-04 | 24.57 | 29.46 | 20.48 | Detected | B4 |
| 2023-04-04 |  |  |  | Not detected | C1 |
| 2023-04-04 |  |  |  | Not detected | TCe |
| 2023-04-04 | 28.94 |  | 28.77 | Detected | B5 |
| 2023-04-04 | 28.71 |  | 28.13 | Detected | B6 |
| 2023-04-04 | 25.34 |  | 22.82 | Detected | C2 |
| 2023-04-04 | 26.42 |  | 24.76 | Detected | TCi |
| 2023-04-11 |  |  |  | Not detected | B1 |
| 2023-04-11 | 26.46 | 26.70 | 24.72 | Detected | B2 |
| 2023-04-11 |  |  |  | Not detected | B3 |
| 2023-04-11 | 29.50 | 29.32 | 26.44 | Detected | B4 |
| 2023-04-11 |  |  |  | Not detected | C1 |
| 2023-04-11 |  |  |  | Not detected | TCe |
| 2023-04-11 |  |  |  | Not detected | B5 |
| 2023-04-11 | 28.22 | 28.29 | 26.00 | Detected | B6 |
| 2023-04-11 |  |  |  | Not detected | C2 |
| 2023-04-11 | 27.04 | 26.47 | 24.71 | Detected | TCi |
| 2023-04-18 | 30.17 | 30.19 | 28.18 | Detected | B1 |
| 2023-04-18 | 28.45 | 29.29 | 27.45 | Detected | B2 |
| 2023-04-18 | 28.34 | 29.04 | 27.14 | Detected | B3 |
| 2023-04-18 | 28.81 | 30.84 | 27.63 | Detected | B4 |
| 2023-04-18 | 29.72 | 29.74 | 28.24 | Detected | C1 |
| 2023-04-18 | 32.11 | 31.12 | 30.88 | Detected | TCe |
| 2023-04-18 | 31.18 | 30.52 | 29.42 | Detected | B5 |
| 2023-04-18 | 29.89 | 29.71 | 27.73 | Detected | B6 |
| 2023-04-18 | 26.42 | 26.50 | 24.99 | Detected | C2 |
| 2023-04-18 | 25.53 | 25.63 | 23.54 | Detected | TCi |
| 2023-04-25 | 28.10 | 29.52 | 26.70 | Detected | B1 |
| 2023-04-25 | 29.06 | 29.17 | 27.57 | Detected | B2 |
| 2023-04-25 | 23.40 | 23.28 | 21.46 | Detected | B3 |
| 2023-04-25 | 29.46 | 30.36 | 28.12 | Detected | B4 |
| 2023-04-25 | 29.80 | 32.17 | 31.62 | Detected | C1 |
| 2023-04-25 | 30.36 | 31.23 | 28.37 | Detected | TCe |
| 2023-04-25 | 28.02 | 28.94 | 26.40 | Detected | B5 |
| 2023-04-25 | 28.18 | 29.80 | 26.25 | Detected | B6 |
| 2023-04-25 | 22.96 | 24.26 | 22.07 | Detected | C2 |
| 2023-04-25 | 26.07 | 27.58 | 25.24 | Detected | TCi |
| 2023-05-02 | 24.57 | 29.46 | 20.48 | Detected | B1 |
| 2023-05-02 | 28.94 | 29.32 | 28.77 | Detected | B2 |
| 2023-05-02 | 28.71 | 28.29 | 28.13 | Detected | B3 |
| 2023-05-02 | 25.34 | 26.47 | 22.82 | Detected | B4 |
| 2023-05-02 | 26.42 | 25.00 | 24.76 | Detected | C1 |
| 2023-05-02 | 26.46 | 26.70 | 24.72 | Detected | TCe |
| 2023-05-02 | 29.50 | 29.32 | 26.44 | Detected | B5 |
| 2023-05-02 | 28.22 | 28.29 | 26.00 | Detected | B6 |
| 2023-05-02 | 27.04 | 26.47 | 24.71 | Detected | C2 |
| 2023-05-02 | 30.17 | 30.19 | 28.18 | Detected | TCi |
| 2023-05-09 | 28.45 | 29.29 | 27.45 | Detected | B1 |
| 2023-05-09 | 28.34 | 29.04 | 27.14 | Detected | B2 |
| 2023-05-09 | 28.81 | 30.84 | 27.63 | Detected | B3 |
| 2023-05-09 | 29.72 | 29.74 | 28.24 | Detected | B4 |
| 2023-05-09 | 32.11 | 31.12 | 30.88 | Detected | C1 |
| 2023-05-09 | 31.18 | 30.52 | 29.42 | Detected | TCe |
| 2023-05-09 | 29.89 | 29.71 | 27.73 | Detected | B5 |
| 2023-05-09 | 26.42 | 26.50 | 24.99 | Detected | B6 |
| 2023-05-09 | 25.53 | 25.63 | 23.54 | Detected | C2 |
| 2023-05-09 | 28.10 | 29.52 | 26.70 | Detected | TCi |
| 2023-05-16 | 29.06 | 29.17 | 27.57 | Detected | B1 |
| 2023-05-16 | 23.40 | 23.28 | 21.46 | Detected | B2 |
| 2023-05-16 | 29.46 | 30.36 | 28.12 | Detected | B3 |
| 2023-05-16 | 29.80 | 32.17 | 31.62 | Detected | B4 |
| 2023-05-16 | 30.36 | 31.23 | 28.37 | Detected | C1 |
| 2023-05-16 | 28.02 | 28.94 | 26.40 | Detected | TCe |
| 2023-05-16 | 28.18 | 29.80 | 26.25 | Detected | B5 |
| 2023-05-16 | 22.96 | 24.26 | 22.07 | Detected | B6 |
| 2023-05-16 | 26.07 | 27.58 | 25.24 | Detected | C2 |
| 2023-05-16 | 28.10 | 29.52 | 26.70 | Detected | TCi |
| 2023-05-23 | 29.06 | 29.17 | 27.57 | Detected | B1 |
| 2023-05-23 | 23.40 | 23.28 | 21.46 | Detected | B2 |
| 2023-05-23 | 29.46 | 30.36 | 28.12 | Detected | B3 |
| 2023-05-23 | 29.80 | 32.17 | 31.62 | Detected | B4 |
| 2023-05-23 | 30.36 | 31.23 | 28.37 | Detected | C1 |
| 2023-05-23 | 28.02 | 28.94 | 26.40 | Detected | TCe |
| 2023-05-23 | 28.18 | 29.80 | 26.25 | Detected | B5 |
| 2023-05-23 | 22.96 | 24.26 | 22.07 | Detected | B6 |
| 2023-05-23 | 26.07 | 27.58 | 25.24 | Detected | C2 |
| 2023-05-23 | 28.10 | 29.52 | 26.70 | Detected | TCi |
| 2023-05-30 | 29.06 | 29.17 | 27.57 | Detected | B1 |
| 2023-05-30 | 23.40 | 23.28 | 21.46 | Detected | B2 |
| 2023-05-30 | 29.46 | 30.36 | 28.12 | Detected | B3 |
| 2023-05-30 | 29.80 | 32.17 | 31.62 | Detected | B4 |
| 2023-05-30 | 30.36 | 31.23 | 28.37 | Detected | C1 |
| 2023-05-30 | 28.02 | 28.94 | 26.40 | Detected | TCe |
| 2023-05-30 | 28.18 | 29.80 | 26.25 | Detected | B5 |
| 2023-05-30 | 22.96 | 24.26 | 22.07 | Detected | B6 |
| 2023-05-30 | 26.07 | 27.58 | 25.24 | Detected | C2 |
| 2023-05-30 | 22.96 | 24.26 | 22.07 | Detected | TCi |
