# Supplementary material for: Multi-scale wastewater surveillance at a Bangkok tertiary care hospital: A potential sentinel site for real-time COVID-19 surveillance at hospital and national levels
Source: PLOS Glob Public Health. 2025 Apr 8;5(4):e0004256. doi: 10.1371/journal.pgph.0004256 (PMC11978038; doi:10.1371/journal.pgph.0004256)
Supplement: S6 Table — (DOCX) [file pgph.0004256.s006.docx]

**S6 Table. Spearman’s Rho Coefficient for the Correlation Between Wastewater Sample SARS-CoV-2 N Gene Ct Values at Various Sample Sites and COVID-19 Clinical Reporting at the Hospital and National Level.** An asterisk indicates Z-test statistical significance after Bonferroni correction.

| **Relationship** | **Sample Site** | **Rho (95% CI)** | **P-Value** |  |
| --- | --- | --- | --- | --- |
| Selected Hospital, Patients | C2 | -0.74 (-0.91, -0.38) | >0.01 | * |
|  | TCe | -0.69 (-0.85, -0.42) | >0.01 | * |
|  | B5 | -0.65 (-0.87, -0.23) | >0.01 | * |
|  | TCi | -0.62 (-0.85, -0.17) | >0.01 | * |
|  | C1 | -0.61 (-0.80, -0.32) | >0.01 | * |
|  | B6 | -0.59 (-0.84, -0.14) | >0.01 | * |
|  | B2 | -0.58 (-0.78, -0.27) | >0.01 | * |
|  | B1 | -0.50 (-0.73, -0.17) | >0.01 | * |
|  | B3 | -0.47 (-0.71, -0.13) | >0.01 | * |
|  | B4 | -0.16 (-0.48, 0.19) | 0.28 |  |
| Selected Hospital, Healthcare Workers | C2 | -0.73 (-0.90, -0.36) | >0.01 | * |
|  | TCi | -0.68 (-0.88, -0.26) | >0.01 | * |
|  | B5 | -0.64 (-0.86, -0.21) | >0.01 | * |
|  | B2 | -0.63 (-0.81, -0.34) | >0.01 | * |
|  | TCe | -0.61 (-0.80, -0.31) | >0.01 | * |
|  | C1 | -0.58 (-0.84, -0.13) | >0.01 | * |
|  | B6 | -0.58 (-0.78, -0.27) | >0.01 | * |
|  | B3 | -0.52 (-0.74, -0.19) | >0.01 | * |
|  | B1 | -0.51 (-0.74, -0.19) | >0.01 | * |
|  | B4 | -0.19 (-0.51, 0.16) | 0.19 |  |
| Thailand | B5 | -0.77 (-0.92, -0.42) | >0.01 | * |
|  | C2 | -0.68 (-0.88, -0.27) | >0.01 | * |
|  | TCi | -0.61 (-0.85, -0.16) | >0.01 | * |
|  | C1 | -0.60 (-0.80, -0.30) | >0.01 | * |
|  | B3 | -0.59 (-0.79, -0.29) | >0.01 | * |
|  | B2 | -0.54 (-0.76, -0.22) | >0.01 | * |
|  | TCe | -0.50 (-0.73, -0.17) | >0.01 | * |
|  | B6 | -0.45 (-0.77, 0.04) | 0.02 |  |
|  | B1 | -0.42 (-0.68, -0.08) | >0.01 | * |
|  | B4 | -0.11 (-0.44, 0.24) | 0.46 |  |
