# Supplementary material for: Multi-scale wastewater surveillance at a Bangkok tertiary care hospital: A potential sentinel site for real-time COVID-19 surveillance at hospital and national levels
Source: PLOS Glob Public Health. 2025 Apr 8;5(4):e0004256. doi: 10.1371/journal.pgph.0004256 (PMC11978038; doi:10.1371/journal.pgph.0004256)
Supplement: Attachment — Submitted filename: Response to Reviewers.docx [file pgph.0004256.s007.docx]

**Comments to the Author**

1. Does this manuscript meet PLOS Global Public Health’s [publication criteria](https://track.editorialmanager.com/CL0/https:%2F%2Fjournals.plos.org%2Fglobalpublichealth%2Fs%2Fcriteria-for-publication/1/010f0193d3535064-e6ca2fc4-9fb1-431c-8b3b-e37835e03ded-000000/C85VcFdAoSoB6n7FPrHy8WNEcGkXXVoO1hVmZAUCnlU=189)? Is the manuscript technically sound, and do the data support the conclusions? The manuscript must describe methodologically and ethically rigorous research with conclusions that are appropriately drawn based on the data presented.

Reviewer #1: Partly

Reviewer #2: Yes

Reviewer #3: Partly

2. Has the statistical analysis been performed appropriately and rigorously?

Reviewer #1: Yes

Reviewer #2: Yes

Reviewer #3: Yes

3. Have the authors made all data underlying the findings in their manuscript fully available (please refer to the Data Availability Statement at the start of the manuscript PDF file)?

The [PLOS Data policy](https://track.editorialmanager.com/CL0/https:%2F%2Fjournals.plos.org%2Fglobalpublichealth%2Fs%2Fdata-availability/1/010f0193d3535064-e6ca2fc4-9fb1-431c-8b3b-e37835e03ded-000000/HJ_CWnQsGWMSEEdacz1p7DFpJyoN4Q_C9eKvPEovuFE=189) requires authors to make all data underlying the findings described in their manuscript fully available without restriction, with rare exception. The data should be provided as part of the manuscript or its supporting information, or deposited to a public repository. For example, in addition to summary statistics, the data points behind means, medians and variance measures should be available. If there are restrictions on publicly sharing data—e.g. participant privacy or use of data from a third party—those must be specified.

Reviewer #1: Yes

Reviewer #2: Yes

Reviewer #3: Yes

4. Is the manuscript presented in an intelligible fashion and written in standard English?

PLOS Global Public Health does not copyedit accepted manuscripts, so the language in submitted articles must be clear, correct, and unambiguous. Any typographical or grammatical errors should be corrected at revision, so please note any specific errors here.

Reviewer #1: Yes

Reviewer #2: Yes

Reviewer #3: No

5. Review Comments to the Author

Please use the space provided to explain your answers to the questions above. You may also include additional comments for the author, including concerns about dual publication, research ethics, or publication ethics. (Please upload your review as an attachment if it exceeds 20,000 characters)

Reviewer #1: Dear Editor,

I have reviewed the manuscript "Multi-scale wastewater surveillance at a Bangkok tertiary care hospital: A potential sentinel site for real-time COVID-19 surveillance at hospital and national levels" and provide my detailed assessment below.

OVERALL ASSESSMENT
This manuscript presents a valuable contribution to wastewater-based epidemiology, particularly for healthcare settings in regions with limited municipal wastewater infrastructure. The study design is methodologically sound, and the findings have important implications for public health surveillance. While the paper requires some revisions, the core research and conclusions are scientifically valid and worthy of publication.

MAJOR STRENGTHS
1. Novel multi-scale approach examining wastewater surveillance at different spatial resolutions
2. Comprehensive sampling strategy (n=392) over 47 weeks
3. Strong correlation analysis between wastewater data and clinical cases
4. Clear relevance for low- and middle-income countries
5. Well-structured presentation of findings

MAJOR CONCERNS

1. Methodological Details
- RNA extraction efficiency and quality control measures need elaboration
- PCR inhibition controls should be described

Further details were added in both the “Wastewater RNA extraction” and “Real-time RT-PCR SARS-CoV-2 detection” subsections of the Methods section (Lines: 187-188, 211-218). Specifically, we included information on the volume, 5 μl, and contents, lentivirus reference RNA, of the internal control provided by the Fosun COVID-19 RT-PCR Detection Kit (Fosun, Shanghai, China). The internal control was added to each sample prior to the extraction process to evaluate RNA extraction efficiency and ensure that no PCR inhibitors were present during amplification. A cycle threshold (Ct) value of 32 or lower across each of the three fluorescence channels corresponding to the three SARS-CoV-2 genes indicated the validity of the internal control.

- Sample storage/transport conditions require more detail

Further details were added in the “Wastewater sample collection” subsection of the Methods section (Lines: 169-174). Clarification was provided on the use of transportation and storage equipment, temperature monitoring, transport time, and storage conditions. Given that the samples were processed at the same hospital where they were collected, we were able to assure controlled storage conditions and reduce transport time.

- Building occupancy data and its potential impact on results should be discussed
The occupancy type for each sampled building is outlined in Table 1. However, specific data on the number of occupants in each building during the sample collection period is not available. To address the potential impact of occupancy rates on our results, we have incorporated the average annual inpatient bed occupancy rates obtained from hospital records into our analysis. We have added the following statement to the “Study setting” subsection of the Materials and Methods section: “In 2022 and 2023, inpatient bed occupancy rates in the hospital averaged 70.61% and 66.48%, respectively, approaching or falling below the hospital's target rate of 70% for efficient operations” (Lines: 110-112). This inclusion acknowledges the role of occupancy rates in interpreting our study's outcomes.

2. Statistical Analysis
- Multiple testing corrections should be applied

Thank you for these valuable suggestions. To account for multiple comparisons in the correlation analysis, we applied Bonferroni corrections. The hypothesis of the Spearman’s rank correlation test was to evaluate the association between wastewater samples and clinical case data across each sample site in the selected hospital. Each sample site was considered independently, leading to three tests for our hypothesis and a Bonferroni-adjusted alpha value of 0.017 (0.05/3). A Fisher Z-Transformation was used to calculate Bonferroni-adjusted 95% confidence intervals, following the methods of Bonnet & Wright (2000). Further details on this approach are provided in the “Data Analysis” subsection of the Materials and Methods section (Lines: 240-242).

- Time-series analysis would strengthen temporal correlations

Thank you for your insightful comments and the suggestion to perform a time series analysis adjustment for the correlation between semi-quantitative cycle threshold (Ct) values and clinical cases. We appreciate your interest in addressing potential correlation inflation due to autocorrelation within our analysis. After consideration, we believe that typical transformations to achieve stationarity, such as differencing, may not be suitable for the semi-quantitative Ct values utilized in our study. Consequently, we have chosen to employ Spearman’s rank correlation, which, while not assuming linearity, effectively captures the monotonic relationship inherent in our wastewater Ct values. We are grateful for your feedback and remain open to further suggestions for improvement.

- Power analysis should be included

Thank you for the suggestion. Power was calculated post-hoc using the Fisher Z transformation with a rho_H0_ = 0 and rho_H1_ = 0.65 based on wastewater correlation in Thailand with community case findings from Sangsanont et al. (2022). The alpha value underwent Bonferroni’s correction consistent with analysis in the study. Power was found to be 0.892 and 0.996 for samples sites with a sample size of 26 and 48, respectively. Given that the power calculation is post-hoc I ask that the power analysis is provided in this response for curiosity sake and not included in the manuscript.

- Confidence intervals for correlation coefficients needed

This point is addressed above in response to multiple testing corrections.

- Outlier analysis and sensitivity testing warranted
Thank you for the suggestion for an outlier analysis. In Fig 4, the box plot of N gene Ct values by sample site uses the IQR method to visually depict outliers for each sample site. We stated “N gene Ct value outlier values were retained for analysis due to expected natural variation and shifts in pathogen prevalence” (Lines: 309-310). Based on our analysis and interpretation of results, we do not believe that outliers indicate measurement error including sampling or processing issues and reflect fluctuations in contributions to the hospital wastewater. Upon further clarification of “formal outlier analysis” we are willing to conduct additional analyses.

Additionally, thank you for your insightful comment regarding the potential use of sensitivity analyses in our study. We acknowledge their value in evaluating the robustness of findings and validating predictive models in wastewater research. However, given the scope and data availability of our study, conducting a sensitivity analysis is challenging and may not yield meaningful insights at this stage. Our primary objective is to explore the presence of a monotonic relationship between clinical data and sampling sites within a Thai tertiary hospital. While developing a more intricate predictive model to address this question would be advantageous, our existing data set, evidenced by the Spearman's correlation analyses, reflects our current capacity for data collection. We recognize that future studies would benefit from incorporating sensitivity analyses. To facilitate this, additional data collection will be critical, specifically gathering comprehensive information on the number of viral copies per sample, the number of patients in each building, and detailed flow rate and volume data at each sample site during the time of collection. These enhancements would enable the development of a robust model to more accurately predict the number of infected individuals within specific hospital buildings or clusters.

3. Data Availability
- Raw RT-qPCR data should be provided

Raw RT-PCR data was made available. The following statement in the “SARS-CoV-2 RNA detection and quantification in wastewater samples” subsection of the Results section was added to reflect this, “Raw RT-PCR wastewater data is made available in the S3 Table” (Lines: 300-301).

- Quality control metrics need inclusion

We hope that the concerns about quality control metrics were addressed in our responses to the Materials and Methods section above.

- Building-specific metadata would be valuable

Thank you for this comment. Table 1 includes all the building-specific metadata and demographics that were collected and accessible for this study.

- Database accession numbers for genomic data required

Thank you for this suggestion. We have added the GenBank and GISAID accession numbers from the selected hospital to the supplemental file in the S2 Table along with a statement detailing this in the “Clinical COVID-19 trends from the selected hospital and the greater community” subsection of the Results section (Line: 280). In this process, 4 of the 209 genomic samples were below the minimum standards required for both databases leading to their removal from our analysis. Fig 3 was updated to reflect this.

SPECIFIC RECOMMENDATIONS

Methods Section:
1. Add details about:
- RNA extraction controls and efficiency measurements

- PCR inhibition testing protocols
- Sample storage conditions and temperature monitoring
- Transport time between collection and processing

These points were addressed above.

2. Include:
- Building occupancy data if available

This point was addressed above.

- Wastewater flow rate measurements

Thank you for this meaningful suggestion. Flow rate measurements were not recorded at each sample site. However, the building and building cluster wastewater samples were collected from holding tanks prior to their discharge to the hospital's wastewater treatment plant. Due to the low flow rates in these tanks, we opted for grab sampling instead of composite sampling, as is common in local sampling scenarios (Spurbeck et al., 2021). While this approach involves certain trade-offs, it is well-documented and appropriate given the context. Moreover, during the study period, the hospital wastewater treatment center's daily inflow ranged from 2876 to 3558 m³/day (Lines: 143-145). These relatively low fluctuations in daily flow rates suggest similar stable patterns may exist in the building and building cluster holding tanks.

- Temperature and pH measurements of samples

We did not record temperature or pH measurements for our individual wastewater samples. However, we accessed monthly pH data from the influent wastewater at the hospital's wastewater treatment center during the sample collection period. The pH remained relatively stable, ranging from 7 to 8, suggesting consistent conditions across the hospital's wastewater system. While this data provides a general indication of stable pH levels, we acknowledge the importance of site-specific measurements. In future wastewater analyses, we will ensure to record both temperature and pH at each collection site. This will enhance our understanding of potential variations in RNA stability and extraction efficiency and improve the accuracy and reliability of our surveillance efforts.

- Detailed quality control procedures
In response to the request for more detailed quality control procedures, we have added a paragraph in the "Real-time RT-PCR SARS-CoV-2 Detection" subsection of the Methods section. In summary, our results are deemed valid when specific quality control criteria are satisfied. These criteria include: (1) achieving a Ct value of less than 32 for the SARS-CoV-2 positive control across all three targeted SARS-CoV-2 genes, (2) obtaining an undetected Ct value for the negative control in all three SARS-CoV-2 genes to ensure no contamination, and (3) detecting the internal control signal at a Ct value of less than 32 to confirm the efficiency and integrity of the RT-PCR process. Should all three SARS-CoV-2 genes and the internal control be undetected in a sample, the PCR test is repeated to verify the findings. Furthermore, we have included a statement in the "SARS-CoV-2 RNA Detection and Quantification in Wastewater Samples" subsection of the Results section, indicating that all samples met the established quality control criteria.

Results Section:
1. Strengthen by adding:
- Multiple testing corrections for correlations
- Confidence intervals for all statistical measures
- Formal outlier analysis
- Sensitivity analyses
- Time-series analysis of temporal trends
These points were addressed above.

2. Expand discussion of:
- Potential confounding factors

This comment is addressed in the response below pertaining to building usage patterns and clinical testing practices.

- Seasonal effects on viral detection

Thank you for this very helpful comment. The following statement was added to introduce this topic into the Discussion section to promote further investigation into this “At the building and building cluster levels, viral concentration variation was found between sample sites as indicated by median N gene Ct values among positive samples. Variability in the data may be attributed to several factors. […] Seasonal changes in population size and behavior among subgroups of patients and hospital personnel, along with the impacts of shifting weather conditions on wastewater holding tanks, may also contribute to this variability (33, 34)” (Lines: 406-412). This statement is supported by two papers, one detailing a weak correlation between temperature and daily mean viral load and the other describing various mechanisms in which shifts in the weather in Central Thailand may promote COVID-19 transmission.

- Impact of building usage patterns
- Changes in clinical testing practices

Thank you for this important comment. Further clarification was added to the Discussion section to detail this limitation. We added the following statement, “due to the dynamic nature of pandemic-related policies at the selected hospital, such as changes in building usage and testing protocols, there was a lack of consistent records available for these variables. As a result, controlling for these shifts was challenging and may introduce potential bias in our analysis” (Lines: 527-531). We believe that this clarification also more explicitly states the largest uncontrolled confounding factors in our analysis.

Discussion Section:
1. Address:
- Generalizability to other hospital settings

Further clarity was incorporated into the Discussion section to address this limitation. We have now stated the following, “we investigated only one hospital campus, which may not fully represent the diverse resource levels, infrastructure, and patient demographics of other public tertiary hospitals in the region. To better understand the generalizability of our conclusions, further research should include multiple hospital settings, particularly major tertiary care hospitals (1,000+ beds) across Thailand” (Lines: 518-522).

- Cost-effectiveness compared to clinical surveillance

Further detail was added to the Discussion section to support our claim of cost-effectiveness. We added the statement, “Integrating WBE into hospital infectious disease sentinel surveillance systems can enhance clinical monitoring while reducing costs. During the study period, the cost for RT-PCR testing was $40 per individual clinical sample, compared to $45 for each pooled wastewater sample from multiple patients. This highlights the economic benefits of using wastewater data for complimentary surveillance alongside clinical testing, particularly in LMIC settings” (Lines: 484-488).

- Limitations of the approach

Thank you for this comment. We have expanded the existing limitation paragraph at the end of the discussion to address this.

- Practical implementation considerations
I hope the revisions in this draft adequately address practical implementation considerations. Specifically, the inclusion of costs for clinical and wastewater samples at our selected hospital, along with detailed limitations on the generalizability of our findings in the Discussion section, are intended to assist future researchers in evaluating the feasibility and uncertainties involved in establishing a multi-scale hospital wastewater surveillance system. As well, a reference to a study in Chicago that detected mpox in wastewater at healthcare facilities prior to clinical reporting was added to further demonstrate the expansions of wastewater surveillance to other pathogens within clinical environments (Lines: 502-504).

2. Include:
- Recommendations for sampling frequency

Determining the optimal sampling frequency was not within the original scope of our study. The study's weekly sampling frequency, determined by budget and labor constraints, may not capture the optimal frequency for wastewater surveillance. Potential statistical explorations are limited by this sampling rate. A statement was added to the Discussion section to reflect this, “our sampling schedule restricted us to once-per-week collection, limiting our ability to assess the benefits of more frequent sampling. Although our wastewater data indicated similar detection trends across sample sites at this frequency, this pattern did not persist during periods of low reported COVID-19 cases (February to early March 2023). This suggests that more frequent sampling may be beneficial for capturing trends when a disease is rare or emerging” (Lines: 522-527). Providing the costs for wastewater and clinical sampling aims to help future researchers explore more frequent sampling within their budgetary constraints.

- Guidelines for site selection

The following statement was added to the Discussion section, “Ten sample sites were selected to capture diverse populations and wastewater matrices, while also considering budget constraints and hospital permissions” (Lines: 381-383).

- Quality control requirements

The following statement was added to the Discussion section to summarize the quality controls utilized in this study, “Reliable SARS-CoV-2 detection in wastewater samples was ensured through stringent quality control measures in this study, including positive and negative controls, and an internal control to verify RNA extraction efficiency. Furthermore, the sampling, transportation, and storage procedures were carefully managed to preserve sample integrity. These comprehensive measures contributed to the consistency and validity of the results reported throughout the investigation” (Lines: 511-516).

- Resource implications

Thank you for this suggestion. Further details on the guidelines for site selection were included as detailed in our response above (Lines: 381-383). Additionally, as stated in the response to the cost-effectiveness comment, further detail was added pertaining to the cost per wastewater sample in Thailand.

- Add new figure showing temporal trends with confidence intervals
Thank you for your valuable feedback. In response to your request to add a new figure showing temporal trends with confidence intervals, we have updated Figure 6 to include Bonferroni-adjusted 95% confidence intervals. This addition is intended to provide a more comprehensive representation of uncertainty in our analysis without losing the spatial resolution of our findings.

MINOR ISSUES
1. Editorial:
- Some redundancy in methods section

Thank you for the feedback. We have attempted to address this issue to the best of our abilities by deleting and combining redundant statements. If this comment is in reference to Table 1, our hope was to provide the sample site metadata and demographic information in both written and tabular forms for clarity.

- Inconsistent abbreviation use

Thank you for catching this. When referring to the hospital treatment of wastewater, we have switched to referring to it as a wastewater treatment center to be more consistent with the “TC” label used for the influent and effluent sample sites. Additionally, “RT-PCR” has been replaced with “RT-PCR” to be more consistent with the names of the kits.

- Variable formatting of units

Again, thank you for catching this. The following changes were made. First, cycle threshold was defined at its first use. Additionally, “N gene Ct value” replaced “Ct value” in each case where appropriate. Second, a space was emitted prior between the number and “°C”. Finally, “μl” was replaced with “μL” at a single location.

- References need updating

Thank you for this note. We have refiled all our references into EndNote using RIS files from the online publications and used the Vancouver preset to generate citations. Additionally, all article titles have been updated for consistency in capitalization. We hope that these changes address your concerns.

2. Clarity:
- Better define building categories

Additional detail to the building category statement has been added in the “Study setting” subsection of the Materials and Methods section. The following statement now is included, “Sample sites included various configurations of building wastewater systems: six standalone buildings, each with independent wastewater tanks (designated as B1 to B6); two clusters, each consisting of three buildings sharing a common wastewater tank (designated as C1 and C2); and both influent (TCi) and effluent (TCe) samples from the hospital's wastewater center” (Lines: 120-124).

- Explain sampling time selection

Thank you for the comment. In the “Wastewater sample collection” subsection of the Materials and Methods section the following statement was added, “For each sample site, 250 mL grab samples were collected with a stainless-steel sampling dipper between 09:30 AM and 10:30 AM. Samples were collected in the morning to effectively capture human contributions to the wastewater systems, aligning with facility opening times and typical daily human activity patterns” (Lines: 164-168). While debate remains over the best time to collect wastewater samples, the general practice observed by wastewater studies is to collect in the morning to capture the “morning flush.”

- Clarify quality control criteria

This point was clarified and addressed in the comments above.

- Expand on statistical methods

This point was addressed in the comments above.

RECOMMENDATIONS FOR AUTHORS
1. Provide complete methodological details
2. Strengthen statistical analysis
3. Include all raw data and quality control metrics
4. Expand discussion of limitations
5. Address practical implementation considerations

PUBLICATION RECOMMENDATION
Accept with Major Revisions

The manuscript presents valuable research with important implications for public health surveillance. The suggested revisions would strengthen the paper's scientific rigor and utility to the field. The authors should:
1. Address all methodological concerns
2. Strengthen statistical analysis
3. Provide complete data
4. Expand discussion of limitations and practical implementation

With these revisions, this paper would make a contribution to the literature on wastewater-based epidemiology and would be suitable for publication in PLOS Global Public Health.

Reviewer #2: The research on multi-scale waste water surveillance at a Bangkok tertiary hospital added another insight into the relevance of environmental surveillance as another pathway to early detection of some pathogenic organisms such as SARS Cov-2 in waste water and this could improve onsurveillance of other water-borne diseases. The study was well thought out and well implemented. The language was scientific and easy to understand. The use of statistical packages to support findings is okay. The conclusion was direct at the objectives.

Thank you for your meaningful and supportive feedback.

Reviewer #3: The abstract needs to be written in an organized and unambiguous manner.

Thank you for this very helpful feedback. Significant changes have been made to the abstract. This includes organizing my thoughts sequentially from background to methods to results to the study’s conclusions. Additionally, further detail has been added to provide less ambiguity.

113: “Central location” should be changed to centrally located

Thank you for this grammatical correction. This change has been made in both uses of “central location” in the manuscript.

134: There is fig. 1 without the provision of such figure

Thank you for your comment. We have verified that our initial submission includes a file titled "Fig_1," which accurately depicts the figure referenced in the paper. Please inform us if the issue persists, and we will promptly address it.

The study design is not cleared
Ethical approval was not obtained from the IRB of the hospital before the collection of clinical data

Thank you for this note. The following statement has been added to the “Clinical COVID-19 case data sources” subsection of the Materials and Methods section, “Clinical data used in this study were anonymous and approved by the Institutional Review Board of the Faculty of Medicine, Chulalongkorn University (IRB. No.221/65)” (Lines: 231-233).

6. PLOS authors have the option to publish the peer review history of their article ([what does this mean?](https://track.editorialmanager.com/CL0/https:%2F%2Fjournals.plos.org%2Fglobalpublichealth%2Fs%2Feditorial-and-peer-review-process%23loc-peer-review-history/1/010f0193d3535064-e6ca2fc4-9fb1-431c-8b3b-e37835e03ded-000000/BeZV1ew2rqi5_J8BNrSYFTy7OMkL1Ir2LjUYlCy0ozM=189)). If published, this will include your full peer review and any attached files.


**Do you want your identity to be public for this peer review?** If you choose “no”, your identity will remain anonymous but your review may still be made public.


For information about this choice, including consent withdrawal, please see our [Privacy Policy](https://track.editorialmanager.com/CL0/https:%2F%2Fwww.plos.org%2Fprivacy-policy/1/010f0193d3535064-e6ca2fc4-9fb1-431c-8b3b-e37835e03ded-000000/FRLr5SVFmKpArzA7pzIPZED9aJrjqftoDWEAmscbZx0=189).

Reviewer #1: No

Reviewer #2: No

Reviewer #3: No
